# On the Effects of the Heating Treatment of Burns and Scalds

**Published:** 1806-04

**Authors:** 


### *On the* Effects *of the* heating Treatment *of* Burns *and* Scalds. *By* Dr. Kinglake.

IN the 81st Number of your Journal, is a paper by Dr. Kentish, containing observations in favour of the stimulant treatment of burns and scalds, in my opinion not sufficiently qualified to exempt them from comment.

The Doctor, after speaking with unbounded confidence of the curative powers of the treatment he recommends, adds, "for my own part, I had arrived at that certainty of prognosis, that I could have insured the life of an individual by the treatment I recommended, and his death by any other." This opinion, under any circumstances, is surely too absolute for the precarious issues of medical practice.

It is not my purpose so much to question the correctness of Dr. Kentish's confidence in the efficacy of his terebinthinate remedy, in the case of burns and scalds, as to object to its being the only alternative from death in difficult cases. Stimulant applications have always appeared to me to be unsuitable in circumstances of inflammatory excitement, however induced, and more particularly so, when resulting from the destructive violence of fire. My objecting therefore to a proposed remedy, which neither principle nor occasion had ever led me to subject to trial, cannot be justly imputed to irrational prejudice. The alleged benefits of the practice are, in my judgment, superfluous, and directly repugnant to my accustomed mode of rendering the most desirable relief.

An early and unremitted application of cold water has, in numerous instances of burns and scalds, under my direction, afforded every aid that either the patient or practitioner could reasonably require. It has arrested the inflammatory career of mischief, and obviated those painful and ulcerative consequences, which are said to be so certainly within the salutary controul of oil of turpentine. The symptomatic fever which speedily ensues the unmitigated torture of burns and scalds, is effectually prevented, by keeping the injured part, during a few hours, under the influence of water, at the atmospheric temperature. It does more, it often either entirely rescues the part from vesication, or if that mischief happens, it is merely limited to the scarf skin. The true skin is not involved in ulcerative inflammation, and consequently the tedious processes of suppurating, sloughing, incarning, and cicatrizing catrizing are superseded; what can the stimulant treatment effect more? or rather, how can it effect so much? Dr. Kentish does not inform us. He refers to experience, an authority to which all must bow assent, when that experience has been unquestionable.

If a patient, writhing under a degree of agonising torture from an extensive burn or scald, that *must*, agreeably to Dr. Kentish's "prognosis," necessarily terminate in death by any other mode of treatment than the fiery one which he recommends, be the *ground* on which we are to repose our faith in its *singular* and *infallible* powers; the Doctor must allow me to doubt the accuracy of his "prognosis," and to object to the boasted superiority of his remedy.

If both heat and cold prove remedial in the same complaint, the strange discordancy would involve the paradoxical question, which of the two opposite agents is the more salutary? This seeming contrariety of effect has not been explained by Dr. Kentish, in the paper alluded to; but it has since been undertaken by Mr. Harrold, in the last (84th) Number of your Journal. The well known Brunonian doctrine of indirect debility, affords the solution of the problem, which Mr. Harrold offers to the public.

To apply this undoubted and important physiological principle to the case in discussion, Mr. Harrold insists, that a degree of violence, approaching to that of burning, must be continued to cure the injury sustained; or, in other words, that though destructive stimulation has been afflicted, a somewhat slighter degree of the same agency is necessary to reconduct the powers of life to the healthful standard. The untenableness of this position may be aptly compared to the conduct of a horseman, who after urging his horse, by lashing and spurring, to an ungovernable speed, would be absurd enough to attempt to restrain him, by continuing to inflict nearly an equal degree of the same stimulant violence. Would not the irritated animal rather pursue his course with accelerated velocity, than resume a temperate pace under such goading discipline? Does not the parallel strictly hold with respect to the inconsistency of exasperating the inflammatory effects of fire, by applying to the burnt part additional excitement? What constitutes the diseased state of an inflamed part from the agency of fire? Is not every fibre capable of increased action, suffering under a most painful degree of inflammatory violence? Is there any conceivable want of excitement in such such a state that could be salutarily supplied by either oil of turpentine or any other stimulant article? Do not the attending torment, the fiery aspect of the mischief, and common sense, concur in condemning the incongruity of aggravating the morbid state, by continuing the stimulant violence which induced it.

The theory which prescribes the re-conducting of inflammatory disease to the standard of health, by only gradually diminishing the force of the violence which occasioned it, is, in my apprehension, fraught with gangrene and death. It would appear to have been derived from licentious speculation; and though it may be plausible on paper, and have its hypothetical votaries, yet its practical application must necessarily involve a responsibility, much too serious, for my dread of its danger to encounter.

If morbid excess of stimulant power were to be thus measured back to the healthful state, what justifiable intention of cure has the medical practitioner to pursue, in endeavouring to reduce the incipient violence of febrile and inflammatory affections, by withholding the usual application of exciting agencies? If, pursuant to the Brunonian doctrine of reducing excitement, only by a slow series of diminution be the correct method of treating diseases of indirect debility induced by excessive stimulation, when can a warranty arise for copious bleeding, purging, perspiration, and for a rigid abstinence from all alcoholic or fermented liquors? Under no circumstances whatever. The diseases of direct debility will imperiously demand liberal excitement, whilst those which result from an indirect failure of strength, or those descriptively, from excessive action of motive power, are to be combated with a degree of excitement approaching to that which occasioned the morbid excess.

The whole of this doctrine appears to proceed on erroneous notions of the nature and effects of vital action, and of its salutary and morbid modifications. The error seems to originate in mistaking the *quantity* for the *quality* of excitement in the several states of life, health, and disease. The motive power of life is connected with peculiar organization, and the various appropriate influences exerted on it by suitable stimulants. The perfection of this agency is health, its default disease. In investigating the ground of this default, the object to be determined is the quality of the deviation from health by which which it is constituted, and not the excessive or deficient force by which it is characterised.

The abstract question of too much or too little action is a mere hypothetical reverie compared with the notable fact, that every departure from the healthful state is necessarily more or less stimulant. It is this morbid change that has substituted irregular excitement for the orderly movements of health, and it is this irregular excitement, that becomes the leading object of attention, in the cure of diseases. An inflammatory affection of the brain occurring in the unbroken strength of youthful health, and the paralytic affection of the aged, arising from the diminished energies of life, are both marked, in a considerable degree, by morbid excess of action. In both, the cheek assumes the changeful flush, and the pulse the agitated throb, though with unequal violence and duration, and in both a guarded procedure with stimulant treatment is necessary. The morbid excitement in both states incessantly diminishes vital energy; in the former case with a healthful, in the latter with a mental tendency. Hence inflammatory disorders often gradually wear out the morbid excitement, and are thus spontaneously cured; whilst those of diminished energy, exhaust the remains of life, and terminate in death. Every variety of disease then is a state of unnatural excitement, and requires to be treated according to the *particular* indication of excessive, or deficient action, and not conformably to any *graduated scale* of stimulation.

Dr. Kentish confesses himself complimented in having the operation of his terebinthinate remedy, likened to the action of one fire extracting another, and observes, that the nearer the agent approaches the nature of fire, without being really such, "the more immediately would it fulfil his intention." Mr. Harrold goes farther, and requires also the co-operative influence of *internal* stimulants, effectually to overcome the local and general affection, ensuing the violence inflicted by burns. With the applauding epithet, "*the beautiful,*" he refers, in support of his opinion, to the Darwinian theory of *reversed sympathy,* which affirms, that in proportion as the excitement of the surface is plus, that of the heart and arteries is minus; and unless the excitement is balanced, there will be great danger of death.

Thus, as well in cases of burns as in the exanthematous affections, (which are in general considered as a salutary transference of internal irritation to the cutaneous surface) the *interior* of the system must be equally excited, lest a *mortal minus* of action should ensue; in other words, a local burn or eruption must be extended through a general affection of equal violence, to obviate a deadly disparity of vital action. This notion is too *electrical*, too much connected with the laws of etherial motion, to be applicable to the functions of animal life.

Burns and all other violent diseases rapidly diminish the energy of vital power, and induce what is denominated indirect debility; but then it should be remembered, that the debility thus produced, is a growing evil under the continued influence of inordinate excitement. The inflammatory action and consequent torture of the burn, necessarily augments the weakness and endangers a mortal decomposition of vital structure, by its unabated continuance. Perchance, additional excitement stopping short at inducing gangrene, may indeed so far exhaust the living power, as to render it incapable of sustaining a painful degree of inflammatory action; hence may ensue, first, an unexcitable state of torpor on the surface of the injured part, and then a lifeless slough, which is ultimately detached by the suppurative inflammation, which recruited strength during the diminution of excitement, had been enabled to supply.

Thus, it would appear, that the most direct and rational mode of affording relief in all inflammatory affections, is not by augmenting the diseased excitement, but by lessening it in such a degree, as to obviate either a mortal, or threatening exhaustion of vital power. Indeed, the main scope of medical surgery, in its treatment of inflammatory and ulcerative surfaces, would seem to consist in avoiding additional excitement, and in reducing that which is inseparably connected with those diseased states, within such limits, as may at once prevent farther diminution of vital power, and allow the innate disposition to recover the healthful action by an uninterrupted procedure.

*Taunton, Feb.* 25, 1806.

